# Correlation between the microinflammatory state and left ventricular structural and functional changes in maintenance haemodialysis patients

**DOI:** 10.3892/etm.2013.1131

**Published:** 2013-05-28

**Authors:** LIHUA SHI, JIE SONG, XIAODONG ZHANG, YING LI, HUI LI

**Affiliations:** Department of Nephrology, Affiliated Hospital of Logistics College of Chinese People’s Armed Police Forces, Tianjin 300162, P.R. China

**Keywords:** haemodialysis, microinflammatory, left ventricle, structural and functional changes

## Abstract

The aim of this study was to examine the correlation between the microinflammatory state and structural and functional changes of the left ventricle in maintenance haemodialysis patients (MHD). In total, 48 MHD patients and 30 healthy volunteers participated in this study. The microinflammatory state was detected from high-sensitivity C-reactive protein (hs-CRP), interleukin-6 (IL-6) and tumour necrosis factor-α (TNF-α) levels determined by ELISA. The structure and function of the left ventricle was measured according to ultrasound cardiogram examination. The serum levels of hs-CRP, IL-6 and TNF-α in the MHD patients were higher compared with those in the controls (P<0.05). Furthermore, the measurements of the left atrial diameter (LAD), left venticular diameter (LVD), interventricular septal thickness (IVST), left ventricular posterior wall thickness (LVPWT) and the left ventricular mass index (LVMI) increased significantly and the left ventricular function (LVEF) was reduced. Correlation analysis demonstrated that the concentrations of hs-CRP, TNF-α and IL-6 correlated with the LVMI (P<0.05), but only hs-CRP correlated with the loss of function of the heart in the haemodialysis patients (P<0.05). The microinflammatory state may be closely associated with the structural and functional impairment of the heart in MHD patients.

## Introduction

The microinflammatory state occurs when, in the absence of overt clinical signs in cases of infection, the body is affected by a variety of microorganisms, endotoxins, chemicals and circulating immune complexes (CICs) which are formed from the integral binding of an antibody to a soluble antigen. These act as stimuli for proinflammatory factor release as the focus of the inflammatory response, and result in the systemic circulation of inflammatory proteins and increased levels of inflammatory factors. In patients with uremia, the microinflammatory state exists generally, and for maintenance haemodialysis (MHD) patients it is particularly apparent. The influential factors are: i) the uremic symptom state; ii) dialysis membrane biocompatibility; iii) dialysate contamination; iv) oxidative stress; and v) vascular access and infection (1–4).

Microinflammation is a major cause of atherosclerosis in patients with end-stage renal disease (ESRD). Previous studies have revealed that cardiovascular disease (CVD) is the most common cause of mortality in dialysis patients, with ∼40–50% of ESRD patients succumbing to it (5–7). Silva *et al* examined the autopsies of 160 uremic patients and identified that 91% of patients with varying degrees of myocardial interstitial fibrosis and cardiac weight were aggravated by prolonged dialysis. Left ventricular hypertrophy (LVH) and left ventricular diastolic dysfunction were the most prominent features of uremic myocardial damage and are the independent factors that affect the survival rate of ESRD patients (8).

Atherosclerosis is a causative factor for CVD and a major complication that determines the morbidity and mortality in dialysis patients. For dialysis patients, atherosclerosis is common and the causative factors are complicated. A study by Silva *et al* demonstrated that the most prevalent CVDs in ESRD patients were LVH, atherosclerosis, valvular disease and coronary artery disease, and that hypertension and dyslipidemia were common risk factors associated with CVD (8).

A considerable number of factors are involved in the progression of CVD in ESRD patients, including inflammation, vascular calcification and LVH. Inflammation markedly predicts cardiovascular mortality rates among dialysis patients. Microinflammatory factors are important in the process of atherosclerosis and left ventricular remodeling in dialysis patients, and promote pathological processes (8–12). Inflammation is critical in the evolution of atherosclerosis (13,14).

The microinflammatory state is evidenced by elevated circulating levels of C-reactive protein (CRP), interleukin (IL)-6, high-sensitivity C-reactive protein (hs-CRP) and tumour necrosis factor-α (TNF-α), all of which are considered to be good predictors of a poor outcome (15–19). It has been reported that the relative risk associated with each 1 pg/ml increase in the concentration of IL-6 was increased by 4.4% in ESRD patients. It has also been demonstrated that low circulating levels (<2 pg/ml) of IL-6 are sufficient to activate certain inflammatory pathways and differentially regulate gene expression (12).

In patients with ESRD, serum CRP is a good predictor of mortality and adverse cardiovascular events (20). Hs-CRP is considered to be an acute phase reactant, but is also produced in smooth muscle cells within coronary arteries and is preferentially expressed in diseased vessels (21). Hs-CRP is a marker for low-grade sustained inflammation and its serum concentration inversely correlates with plasma n-3 fatty acid levels (17). TNF-α is a pro-inflammatory cytokine previously reported to have damaging effects on various body tissues, particularly in chronic renal failure (CRF) patients (19). Increased levels of IL-6 and TNF-α have been associated with vascular disease in the general population in addition to dialysis patients (2).

The microinflammatory state of ESRD is accompanied by left ventricular remodeling (20). However, the exact roles of hs-CRP, IL-6 and TNF-α in the remodeling process are unknown. Therefore, in the current study, the microinflammatory state, as evidenced by increased levels of hs-CRP, IL-6 and TNF-α, was detected in order to demonstrate its role in the remodeling process.

## Patients and methods

### Patients

Forty-eight haemodialysis patients were enrolled in this study (25 males and 23 females). The patients were aged between 26 and 45 years old (mean, 41.30±8.90 years) and had undergone dialysis for 1–15 months. The diagnoses varied from chronic glomerulonephritis to renal failure. During dialysis, blood pressures were controlled at 130–140/80–90 mmHg. Fluctuations in the loss of body weight were <5% and haemoglobin levels were >100 g/l. All patients were in a stable condition with no signs of bacterial infection and were observed for almost two weeks. This study was conducted in accordance with the Declaration of Helsinki and was conducted with approval from the Ethics Committee of the Affiliated Hospital of Logistics College of Chinese People’s Armed Police Forces. Written informed consent was obtained from all participants.

In total, 30 healthy volunteers (17 males and 13 females, aged 25–48 years old) were used as controls in this study. The healthy volunteers also provided informed consent.

### Dialysis

The patients underwent dialysis three times a week, for 4 h each time. A disposable polysulfone membrane dialyzer (Fresenius Model F7-HPS 1.5M2, Borkenberg, Germany) was used for dialysis. Bicarbonate dialysate and regular heparin anticoagulants were used with a flow rate of 500 ml/min and a blood flow rate of 200–250 ml/min.

### Blood collection and laboratory tests

Blood samples were obtained from the patients prior to haemodialysis and healthy volunteers for physical examination. Following isolation of the serum, the hs-CRP, IL-6 and TNF-α levels were determined using the double antibody sandwich ABC-ELISA method (Boster Biological Co., Wuhan, China).

### Ultrasound echocardiography measurements

Using ultra-sound echocardiography, the left atrial diameter (LAD), left ventricular diameter (LVD), interventricular septal thickness (IVST), left ventricular posterior wall thickness (LVPWT), left ventricular mass index (LVMI) and left ventricular function (LVEF) were investigated for all subjects. In order to maintain consistency, each index was measured in three cardiac cycles and averaged by one doctor. LVMI was calculated using the following equations, defined by Devereux (22), in which: LVMI=LVM/body surface area (BSA), and LVM=1.04 × [(IVST+LVPWT+LVD)^3^−LDV^3^]−13.6.

### Statistical analysis

The Student’s t-test was used to compare measurable indexes, the Chi-square test was used for countable results and P<0.05 was considered to indicate a significant result. Data are presented as the means ± SD. Correlations were calculated using regression methods.

## Results

### Inflammatory changes of haemodialysis patients

Microinflammatory changes were observed in the haemodialysis patients and the levels of hs-CRP, IL-6 and TNF-α in the haemodialysis patients were significantly increased compared with those of the control group.

The demographic and clinical characteristics for the patient population and healthy volunteers are shown in Table I. There were no statistically significant differences between the two groups in terms of gender (P=0.69) and age (P=0.11). The levels of the cytokines Hs-CRP, IL-6 and TNF-α were observed to be significantly higher in the haemodialysis patients than in the control group.

### Structural and functional changes of the left ventricle

Thickening of the left cardiac wall was observed in the haemodialysis patients with reduced heart function. Compared with the control group, the indexes reflecting the structure of the left ventricle including LAD, LVD, IVST, LVPWT and LVMI were increased in the haemodialysis patients. However, heart function was reduced by ∼10% in the haemodialysis patients (Table II). Further analysis revealed that increased levels of hs-CRP, IL-6 and TNF-α correlated positively with the thickening of the cardiac walls (Table III), which may have lead to the reduction in the heart function observed in the haemodialysis patients.

### Hs-CRP, IL-6 and TNF-α levels and left ventricular structure and function changes

According to the normal detection limits in our hospital, a hs-CRP concentration >3mg/l was considered to be elevated and patients with such a hs-CRP concentration were assigned to the increased group. The haemodialysis patients were classified into two groups according to the hs-CRP level: 13 were classified as the normal group and 35 were classified as the increased group. Compared with the measurements in the normal group, the LAD, LVD, LVPWT and LVMI measurements in the increased hs-CRP group were increased whereas the LVEF was significantly reduced. Furthermore, an IL-6 concentration >2.6 pg/ml was set as a cut off limit. In total, 42 patients had an increased IL-6 concentration and 6 patients had normal IL-6 level. Only the LVD and LVPWT increased according to the IL-6 cut off. When the TNF-α level was >3 pg/ml, it was considered to be elevated : 38 patients were thus placed in the increased group and 10 in the normal group. In the increased TNF-α group, the IVST, LVPWT and LVMI were increased significantly and lowered LVEF was observed (Table III).

### Association of inflammatory factors and left ventricular structure and function (r-value) in MHD patients

Correlation analysis revealed that the concentrations of hs-CRP, IL-6 and TNF-α correlated with the structural changes of the left ventricle, as represented by LVMI (P<0.05). In addition to correlating with the structural changes, hs-CRP also correlated with the functional changes of the heart, as represented by LVEF, in which the reduced heart function was accompanied by an increase in hs-CRP levels. However, no correlation was observed between heart function and IL-6 or TNF-α concentration (Table IV).

## Discussion

Microinflammation is significant in the development of atherosclerosis, and the incidence and mortality of cardiovascular events in uremic patients are closely related to the degree of inflammation in the body (8–14). The results of previous studies have demonstrated that hs-CRP, TNF-α and IL-6 levels correlate with coronary heart disease, myocardial depression, atherosclerosis, left ventricular dysfunction and other factors (2,21,23–25).

Microinflammation is the major cause of atherosclerosis in ESRD patients, proving fatal in 9% of cases. Microinflammation during renal failure has been the subject of various studies (5,6). The plasma levels of CRP, hs-CRP, IL-6 and TNF-α are predictors of microinflammation (6–20). However, the level of the microinflammatory state in haemodialysis patients and the role of microinflammatory factors in the process of cardiovascular mortality of patients are not clear. Therefore, in the current study, the plasma cytokines, including hs-CRP, TNF-α and IL-6, were selected as an index of the microinflammatory status. The results demonstrated that the levels of hs-CRP, IL-6 and TNF-α in haemodialysis patients were significantly increased compared with those in the control group. These increases may be one of the reasons for the structural and functional changes observed in the heart.

For haemodialysis patients, CVD is a major complication and a significant cause of mortality. Complications affecting the cardiovascular system have a considerable impact on the survival rate and the quality of life of patients. The current study examined the changes to the cardiac structure and function of haemodialysis patients by echocardiography and discovered that 36 (75%) haemodialysis patients had left ventricular hypertrophy and 12 (25%) had left ventricular systolic dysfunction. Thickening of the left cardiac wall was observed in the haemodialysis patients in addition to the manifestation of a reduction in heart function of ∼10%. The processes leading to changes in the heart are complex, yet they may be explained by the microinflammatory state. When the cut off levels defined by our hospital were considered, significant increases in the levels of hs-CRP, IL-6 and TNF-α combined with changes in cardiac structure and function were observed in the haemodialysis patients. Among the three factors, hs-CRP had the most marked role in the changes of cardiac structure. Our results demonstrated that hs-CRP not only correlated with changes in LVMI but also with changes in LVD and IVST, and reduced cardiac function, consistent with previous reports (10).

In conclusion, the changes in cardiac structure and function may be caused by the microinflammatory state in MHD patients and changes in hs-CRP levels may be an important cause of the changes.

## Figures and Tables

**Table I.** Characteristics of maintenance haemodialysis patients and healthy subjects.

Characteristics	Groups		P-value
	Patients (n=48)	Controls (n=30)	
Gender (male/female)	25/23	17/13	0.69
Age (years)	41.30±8.90	37.70±10.80	0.11
Dialysis period (months)	10.50±7.30	-	-
Hs-CRP (mg/l)	10.33±5.21	1.64±0.52	<0.05
IL-6 (ng/l)	13.88±2.03	1.38±0.35	<0.05
TNF-α (ng/l)	22.68±4.35	10.23±3.86	<0.01

Student’s t-test was used for comparing measurable indices (dialysis period and Hs-CRP, IL-6 and TNF-α plasma levels) and Chi-square test was used for countable results (gender). Data are presented as the means ± SD. Hs-CRP, high-sensitivity C-reactive protein; IL-6, interleukin-6; TNF-α, tumour necrosis factor-α.

**Table II.** Results of echocardiography in maintenance haemodialysis patients.

Parameter	Groups		P-value
	Patients	Control	
LAD (mm)	37.96±7.23	25.41±4.65	<0.05
LVD (mm)	35.67±5.62	31.89±3.95	<0.05
IVST (mm)	14.86±3.25	9.68±1.01	<0.05
LVPWT (mm)	14.01±1.98	7.63±1.21	<0.05
LVMI (g/m^2^)	131.40±22.14	69.03±15.62	<0.05
LVEF (%)	62.98±11.91	73.69±10.46	<0.01

Student’s t-test was used for comparing the index of the left arterial diameter (LAD), left ventricular diameter (LVD), interventricular septal thickness (IVST), left ventricular posterior wall thickness (LVPWT), left ventricular mass index (LVMI) and left ventricular function (LVEF) between the two groups. The data are presented as the means ± SD.

**Table III.** Hs-CRP, IL-6 and TNF-α concentration and the echocardiography index.

Cytokine status	LAD (mm)	LVD (mm)	IVST (mm)	LVPWT (mm)	LVMI (g/m^2^)	LVEF (%)
Hs-CRP (n)						
Increased (35)	39.15±7.53	34.18±6.25	13.96±3.52	15.22±2.56	139.40±20.41	50.14±10.19
Normal (13)	32.14±5.69	28.18±4.23	11.86±2.14	12.67±1.71	103.73±15.62	57.96±5.85
P-value	<0.01	<0.01	>0.05	<0.05	<0.01	<0.05
IL-6 (n)						
Increased (42)	39.23±7.28	35.18±5.83	16.73±5.52	15.96±3.72	138.30±30.59	52.45±11.70
Normal (6)	33.42±5.61	28.33±4.23	12.96±4.07	12.63±1.84	121.85±20.62	59.33±4.86
P-value	>0.05	<0.01	>0.05	<0.05	>0.05	>0.05
TNF-α (n)						
Increased (38)	37.82±5.32	33.81±7.28	15.69±5.25	16.27±3.81	137.90±25.14	50.96±10.25
Normal (10)	34.78±4.29	29.81±3.89	10.97±3.09	13.18±3.01	119.37±12.28	58.76±5.58
P-value	>0.05	>0.05	<0.01	<0.05	<0.05	<0.05

Student’s t-test was used for comparing the index of the left arterial diameter (LAD), left ventricular diameter (LVD), interventricular septal thickness (IVST), left ventricular posterior wall thickness (LVPWT), left ventricular mass index (LVMI) and left ventricular function (LVEF) between the increased and normal groups. The data are presented as the means ± SD. Hs-CRP, high-sensitivity C-reactive protein; IL-6, interleukin-6; TNF-α, tumour necrosis factor-α.

**Table IV.** Correlation of the inflammatory factors and left ventricular structure and function in MHD patients.

Cytokines	LAD (mm)	LVD (mm)	IVST (mm)	LVPWT (mm)	LVMI (g/m^2^)	LVEF (%)
hs-CRP (mg/l)	0.2631	0.4742^a^	0.3631^a^	0.2722	0.4125^a^	−0.3426^b^
IL-6 (ng/l)	0.2415	0.2321	0.2491	0.2473	0.3312^*^	−0.2473
TNF-α (ng/l)	0.3413^b^	0.2312	0.2656	0.2695	0.3397^a^	−0.2752

a Correlation (r) with a significance level of P<0.01;

b correlation (r) with a significance level of P<0.05. MHD, maintenance hemodiaysis; LAD, left arterial diameter; LVD, left ventricular diameter; IVST, interventricular septal thickness; LVPWT, left ventricular posterior wall thickness; LVMI, left ventricular mass index; LVEF left ventricular function; hs-CRP, high-sensitivity C-reactive protein; IL-6, interleukin-6; TNF-α, tumour necrosis factor-α.
